# Cryopreservation of Liver-Cell Spheroids with Macromolecular Cryoprotectants

**DOI:** 10.1021/acsami.2c18288

**Published:** 2023-01-09

**Authors:** Akalabya Bissoyi, Ruben M. F. Tomás, Yanan Gao, Qiongyu Guo, Matthew I. Gibson

**Affiliations:** † Division of Biomedical Sciences, Warwick Medical School, University of Warwick, Gibbet Hill Road, Coventry CV4 7AL, U.K.; ‡ Department of Chemistry, University of Warwick, Gibbet Hill Road, Coventry CV4 7AL, U.K.; § Department of Biomedical Engineering, Southern University of Science and Technology, Shenzhen, Guangdong 518055, China

**Keywords:** macromolecular cryoprotectants, polymers: spheroids, cryopreservation: cell-based assays, macromolecules, ice

## Abstract

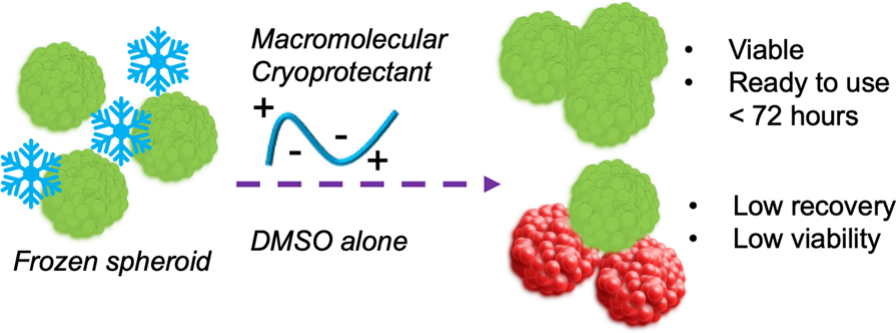

Spheroids are a powerful tool for basic research and to reduce or replace in vivo (animal) studies but are not routinely banked nor shared. Here, we report the successful cryopreservation of hepatocyte spheroids using macromolecular (polyampholyte) cryoprotectants supplemented into dimethyl sulfoxide (DMSO) solutions. We demonstrate that a polyampholyte significantly increases post-thaw recovery, minimizes membrane damage related to cryo-injury, and remains in the extracellular space making it simple to remove post-thaw. In a model toxicology challenge, the thawed spheroids matched the performance of fresh spheroids. F-actin staining showed that DMSO-only cryopreserved samples had reduced actin polymerization, which the polyampholyte rescued, potentially linked to intracellular ice formation. This work may facilitate access to off-the-shelf and ready-to-use frozen spheroids, without the need for in-house culturing. Readily accessible 3-D cell models may also reduce the number of in vivo experiments.

## Introduction

Late-stage failure of drugs is a major challenge in drug discovery, and pre-clinical toxicity can account for up to 70% of attrition.^1^*In vivo* (e.g., rodent) testing is used for screening but does not fully predict human physiological responses.^2^ 2-D cell cultures (monolayers) of liver-derived cells are a key part of the screening process and are suitable for high-throughput and automated screening but, due to the absence of extracellular matrix and cell–cell communication, do not reproduce the *in vivo* niche.^3−5^ Considering this, 3-D (dimensional) models of tissues and organs are emerging, including 3-D cell culture scaffolds, spheroids, and organoids.^3,4,6,7^ Hepatocyte spheroids derived from immortalized and primary cells have been shown to accurately predict *in vivo* toxicity (LD50) for a panel of drugs, but the 2-D equivalents (cell monolayers) did not perform as well.^8^ Despite the strong evidence for their predictive function, spheroids are not widely used in research or discovery programs (relative to 2-D cell models) due to the challenges associated with producing spheroids, including tedious cell culture procedures (>14 days) and variability issues.^9^ At present, spheroids are not routinely cryopreserved, and there are few commercial frozen spheroids that can be simply defrosted and used in screening assays, meaning the individual user must first optimize the preparation processes. Addressing this challenge of easy access would enable the wide use of 3-D cell models and contribute to the goals of reduction, refinement, and replacement for animal testing (3Rs).^10^

Cryopreservation is a platform technology that underpins all biomedical and cell biology research, as well as emerging cell-based therapies,^11−13^ by allowing the banking and distribution of cells and removing the need for continuous culture, which can lead to phenotypic drift,^14^ and is also being resource intensive. For nucleated (mammalian) cells, the most common cryopreservation procedure is based on dimethyl sulfoxide (DMSO) (typically 10%), which protects cells by dehydration, replacing intracellular water, and reducing osmotic shock.^15−19^ Cryopreserving cells in suspension using DMSO typically works well, with >80% post-thaw recoveries possible, but the cryopreservation of more complex models including cell monolayers and, even more so, spheroids remains a major technological challenge. During the freezing of 2- and 3-D cell models, there are challenges of nutrient/cryoprotectant transport to overcome, as well as ice nucleation and propagation across cell–cell contacts.^20,21^ Fine-tuning traditional cryopreservation formulations can improve these outcomes,^19,22,23^ but innovative cryoprotectants are now emerging that can address the damage mechanisms that DMSO does not.^17,24,25^ Cryoprotectants inspired by ice-binding proteins,^26,27^ which can modulate ice growth (recrystallization), have been discovered and applied to cryopreservation.^28−32^ Controlled nucleation (inspired by ice nucleating proteins) has also been shown to benefit 2-D cell monolayer cryopreservation^33,34^ and spheroid cryopreservation.^35^ Vitrification, which uses larger volumes of cryoprotectants (including glycerol and DMSO), can be applied to larger cell models, but the large volume of cryoprotectants is technically challenging to administer uniformly and remove post-thaw. Matsumura and co-workers have introduced polyampholytes (polymers with mixed cationic/anionic side chains) that can dramatically improve cryopreservation outcomes.^36^ Carboxylated poly-ε-lysine has been deployed for the vitrification of several cell types^37,38^ both in suspension and as cell monolayers,^39^ but there are limited structure–property studies on polyampholytes at present.^40,41^ Bailey et al. developed a synthetically scalable polyampholyte, which can be mass-produced on a scale required for cryopreservation: a key consideration is, at 10 wt%, a single 1 mL cryovial would require 100 mg of the new additive.^42^ This polyampholyte is potent, able to cryopreserve stem cells,^43^ red blood cells,^44^ and, crucially, 2-D monolayers, increasing recovery to >90% post-thaw.^45^ DMSO alone only leads to <50% cell recovery of monolayers;^46−48^ hence, existing technology cannot be used to bank cells in their useful format, and the emergence of new cryoprotectants is crucial. Polyampholytes appear to function extracellularly (and hence are easy to remove), aid cellular dehydration, and mitigate osmotic shock.^49^ Polyampholytes have not been applied to the significant challenge of spheroid freezing, to the best of our knowledge.

Here, we report the cryopreservation of hepatocyte spheroids, enhanced by polyampholytes added into a standard DMSO solution. Supplementation with the polyampholyte significantly increases the post-thaw yield and viability of the cells, which are shown to have more intact membranes. The increase in post-thaw cell recovery may be linked to the rescue of actin polymerization post-thaw. Thawed spheroids show toxicological responses equal to fresh (non-frozen) spheroids. These data demonstrate that macromolecular cryoprotectants can be applied to enable off-the-shelf, banked spheroids and will support the wider adoption of 3-D cell models and remove hurdles for new users.

## Experimental Section

### Materials

Poly(methyl vinyl ether-alt-maleic anhydride) with an average *M*_n_ ≈ 80 kDa (416339), tetrahydrofuran, 2-dimethylamino ethanol (471453), Minimum Essential Medium Eagle medium (MEM, M4655), non-US origin fetal bovine serum (FBS, F7524), MEM non-essential amino acid solution 100× (M7145), agarose BioReagent for molecular biology (A9539), Dulbecco’s phosphate-buffered saline (DPBS, D8537), doxorubicin hydrochloride 98% (DOX, 860360), dimethyl sulfoxide Hybri-max, sterile-filtered) (D2650), Triton X-100 (X100), fluorescein-5-isothiocyanate (46950), triethylamine (471283), 0.4% trypan blue (T8154), RNase A from bovine pancreas, Corning CoolCellTM LX cell freezing vial container (CLS432001), and CorningXT CoolSink96F thermoconductive plate (CLS432070) were purchased from Merck (Gillingham, UK). Live/Dead viability assay kit Ethidium homodimer-1 (EI) and calcein-AM (L3224), Corning 96-well white polystyrene microplates (10022561), trypsin (0.25%) and EDTA phenol red (500 mL) (25200072), Invitrogen ActinGreen 488 ReadyProbe reagent (R37110), antibiotic–antimycotic solution 100× (15240062), and cryovials were purchased from Thermo Fisher (Loughborough, UK). Human liver hepatocellular carcinoma cells (HepG2, ECACC85011430) were purchased from ECACC (Salisbury, UK). T-flasks (175 cm^2^) (660175) were purchased from Greiner Bio-One Ltd. A MycoAlert Mycoplasma Detection Kit (LT07-703) was purchased from Lonza (Basel, Switzerland). Micro-mold (12-81-series) was purchased from MicroTissues, Inc. (MA, USA). Spectrum Labs Spectra/Por 2 12–14 kD MWCO (15390762) and Corning 96-Well Clear Ultra Low Attachment Microplates (10023683) were purchased from Fisher Scientific (Loughborough, UK). A WST-1 proliferation reagent was purchased from abcam (Cambridge, UK). Hoechst 33342 was purchased from Life Technologies (CA, USA). TC-treated plates (12 wells), with a lid, (734-2324) were purchased from VWR (Leicestershire, UK). Promega P450-Glo CYP3A4 assay with Luciferin-IPA (V9002) was purchased from Promega (Hampshire, UK).

### Cell Maintenance and 3-D HepG2 Spheroid Formation

Human liver hepatocellular carcinoma cells (HepG2, ECACC85011430) were grown in 175 cm^2^ T-flasks within a humidified incubator at 37 °C and 5% CO_2_ using MEM supplemented with non-US origin FBS (10% (v/v)), MEM Non-Essential Amino Acids Solution 100× (1% (v/v)), and antibiotic–antimycotic solution 100× (1% (v/v)). Mycoplasma contamination was tested routinely with a MycoAlert Mycoplasma Detection Kit (Lonza, Basel, Switzerland).

**Figure 1:**
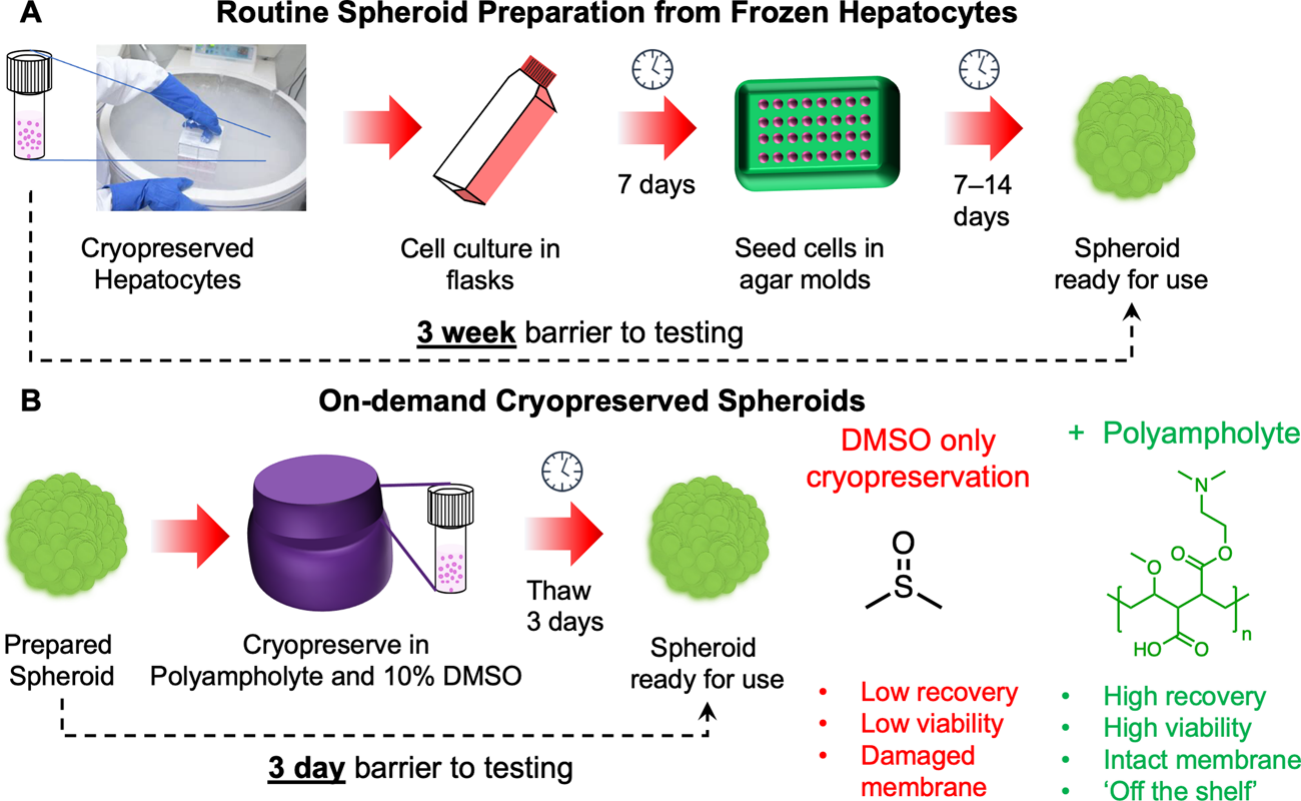
Preparation of spheroids and potential time saved for a user by deploying a macromolecular cryoprotectant (polyampholyte) to enable spheroid banking.^42^ Schematic of the process and time barrier for (A) preparing a spheroid from frozen (suspension) stocks of cells and (B) taking a spheroid direct from cryopreservation.

Spheroid formation: Agarose 3-D structures were fabricated by placing 500 μL of 2% agarose ((w/v) in H_2_O) in each 12-81-series micro-mold (MicroTissues, Inc., Sharon, MA, USA). Following UV sterilization (30 min), the agar structures were placed in a 12-well plate, and 2 mL of complete cell culture medium was added in each well. The plates were placed in an incubator for 1 h. HepG2 cells were subsequently seeded in these structures at a density of 81,000 cells/190 μL (1000 cells per spheroid) or 273,000 cells/190 μL (3375 cells per spheroid) per agar structure for 10 min, allowing the cells to settle (Figure 1). Complete cell culture medium (2 mL) were added in each well, and the plates were placed in an incubator at 37 °C and 5% CO_2_ for 10 days. Cell culture medium was changed every 2 days, and phase contrast images were taken using an Olympus CX41 microscope equipped with a UIS-2 20×/0.45/∞/0–2/FN22 lens to determine spheroid mean diameters. Image analysis was performed using ImageJ software v1.52 (NIH, Bethesda, MD, USA).

### Tracking Spheroid Growth

Spheroids were generated by seeding 273,000 HepG2 cells per agar micro-mold (∼3000 cells per spheroid), as described above. Ten spheroids were selected to monitor spheroid growth by taking phase contrast images daily for 15 days (Olympus CX41 microscope), and ImageJ v1.52 was used to determine the spheroid diameter.

### Cryopreservation Protocol

Cryopreservation of spheroids was carried out using two freezing methods: (i) spheroid freezing in agar micro-molds and (ii) spheroid freezing in cryovials.

(i) Freezing in agar micro-molds: HepG2 spheroids were frozen in the agar structures used to generate them, as described above, in a 12-well microplate. Briefly, following 10 days of spheroid formation, the cell medium was removed and replaced with a cryoprotective agent (CPA) consisting of MEM base media supplemented with 10% (v/v) FBS, 10% (v/v) DMSO, and varying concentrations of the polyampholyte (0–80 mg mL^–1^). Following 10 min of incubation at room temperature (RT), the CPA solution was removed and the 12-well plates were placed on a CorningXT CoolSink96F thermoconductive plate and frozen in a −80 °C freezer overnight. For storage, the vials were placed either in LN_2_ or at 80 °C the following day. Spheroids were thawed in an incubator set at 37 °C and 5% CO_2_ for 5 min.

(ii) Vial freezing protocol: Spheroids generated in agar molds were harvested by inverting the molds into a 12-well plate and centrifuging at 500 RPM for 5 min. Spheroids from each well were placed in 12 cryotubes and frozen in 200 μL of CPA solution consisting of MEM base media supplemented with 10% (v/v) FBS, 10% (v/v) DMSO, and varying concentrations of the polyampholyte (0–80 mg mL^–1^). Following 10 min of incubation at RT, the cryotubes were placed in a pre-cooled (4 °C) Corning CoolCell LX cell freezing vial container and frozen overnight in a −80 °C freezer. Spheroids were removed from the −80 °C freezer and thawed rapidly with a 1 mL of warmed cell culture medium. Spheroids were gently mixed, to reduce thawing time, centrifuged (at 2000 RPM for 5 min), and the supernatant was replaced with 1 mL of complete cell culture media. This washing process was repeated further three times.

### Post-Thaw Spheroid Viability

Three freeze/thaw HepG2 spheroids, frozen using both methods, were transferred into Corning 96-Well Clear Ultra Low Attachment Microplates and incubated with 100 μL of complete growth medium supplemented with 10 μL of WST-1 proliferation reagent (abcam, Cambridge, UK) for 4 h. Optical density (OD) measurements were recorded at 450 and 620 nm (reference wavelength) using a BioTek Synergy HT microplate reader. Non-frozen spheroids were also analyzed using this method to provide a comparative control sample. A blank control consisting of 100 μL of complete growth medium supplemented with 10 μL of WST-1 proliferation reagent with no spheroids was also completed.

### Evaluating Membrane and Nuclear Integrity

Freeze/thaw spheroids, frozen in cryovials as a suspension with 10% DMSO and 0–80 mg mL^–1^ of the polyampholyte, were transferred into a 12-well plate containing a 2% agar pad. After 1 day incubation in an incubator, the spheroids were stained with 2 μM calcein AM, 3 μM ethidium iodide^–1^ (ThermoFisher, Loughborough, UK), and 33 μM Hoechst 33342 (Life Technologies, Carlsbad, CA) in phosphate-buffered saline (PBS). The spheroids were imaged using an FV3000 confocal laser-scanning microscope (Olympus, Tokyo, Japan). Hoechst-stained nuclear material was imaged using a 350 nm diode laser excitation source and 461 nm emission wavelength. Calcein AM and ethidium iodide positive cells were imaged by optical scanning with an argon ion laser excitation source set at 499 and 515 nm wavelengths, respectively, and using 520 nm and 620 nm emission wavelengths.

### Drug-Induced Hepatotoxicity Assays

Freeze/thaw spheroids frozen in cryovials as a suspension in CPA solution containing MEM base media supplemented with 10% (v/v) FBS, 10% (v/v) DMSO, and with or without 20 mg mL^–1^ of the polyampholyte were transferred into a 96-well plate and treated with 0–200 μg mL^-1^ of doxorubicin hydrochloride (DOX, Sigma-Aldrich Corp.). DOX was dissolved in cell culture media. After 24 h of incubation in an incubator set at 37 °C and 5% CO_2_, cell viability was evaluated by a WST-1 assay. DOX-treated spheroids were washed three times before they were incubated with 100 μL of complete growth medium supplemented with 10 μL of WST-1 proliferation reagent (abcam, Cambridge, UK) for 4 h. OD measurements were recorded at 450 and 620 nm (reference wavelength) using a BioTek Synergy HT microplate reader. Non-frozen spheroids were also analyzed using this method to provide a comparative control sample. A blank control consisting of 100 μL of complete growth medium supplemented with 10 μL of WST-1 proliferation reagent with no spheroids was also completed. The test was conducted in triplicate with 10 spheroids with approximately 30k cells in each well of 96-well plates. The IC_50_ values were determined using the sigmoidal concentration-response curve fitting model (Graph Pad, Prism software).

### Cytochrome P450 3A4

Three freeze/thaw spheroids, frozen in cryovials as a suspension with CPA solution containing MEM base media supplemented with 10% (v/v) FBS, 10% (v/v) DMSO, and with or without 20 mg mL^–1^ of the polyampholyte, were transferred into a white opaque 96-well plate. Spheroids were washed either 3-, 5- or 7-days post-thaw with 1× phosphate-buffered saline (PBS; Merck, Gillingham, UK). The PBS was replaced with a culture medium containing a luminogenic CYP substrate, CYP3A4/Luciferin-IPA (3 μM, 50 μL, 1 h) provided by a Promega P450-Glo CYP3A4 assay. The CYP substrate was also added to empty wells as a background measurement. The culture medium containing the CYP substrate (25 μL) was transferred to an opaque white 96-well plate and a luciferin detection reagent (25 μL) was added for 20 min at RT. Luminescence was measured on a Tecan Spark plate reader (Tecan, Switzerland). The CYP activity of non-frozen spheroids was also measured for comparison.

### Cell Cycle Analysis

Cell cycle distributions of the four different CPA treated spheroids were determined by flow cytometry using propidium iodide (PI) DNA staining.^2^ Briefly, freeze/thaw HepG2 spheroids, frozen using both methods with CPA solutions containing MEM base media supplemented with 10% (v/v) FBS, 10% (v/v) DMSO, and with or without 20 mg mL^–1^ of the polyampholyte, were treated with trypsin (0.25%) and EDTA (1 mM) for 5 min. The harvested cells were washed with PBS three times and 1 × 10^6^ cells were resuspended in cold 70% ethanol (1 mL) for 30 min at 4 °C. Cells were washed with PBS and incubated in a solution of PBS containing 20 μg mL^–1^ PI and 100 μg mL^–1^ of RNase A for 30 min. Non-frozen spheroids were also stained for comparative cell cycle measurements. Flow cytometry was performed on a BD Accuri C6 using a 488 nm excitation source and a 585/40 filter. BD CSampler Plus software (v 1.0.34.1) was used for data collection and processing. Cell cycle analysis was completed using FlowJo (Tree Star Inc.).

### Statistical Analysis

GraphPad Prism 5.0 software was used to analyze the data. To determine significance between the means of the two groups, an unpaired two-sided *t*-test was used.

## Results

Spheroids (and 2-D monolayers) are challenging to cryopreserve with conventional DMSO-only solutions. To investigate if a macromolecular cryoprotectant (structure in Figure 1)^42,45^ could address the limitations of DMSO-only freezing, HepG2 (hepatocarcinoma epithelial) spheroids were chosen as a representative cell type. HepG2 cells are widely used in toxicological screening^5,50^ and HepG2 spheroids have been validated to predict in vivo toxicological outcomes.^8^ Here, spheroids were prepared using an agarose micro-mold technique, enabling precise control over spheroid dimensions, Figure 1A. Images of prepared spheroids and growth curves are in the Supporting Information, Figure S1. Spheroid preparation requires 2–3 weeks, the major caveat preventing their widespread use. We hypothesized that on-demand spheroids, ready to use in 3 days, can be generated through cryopreservation with both DMSO and macromolecular cryoprotectants, specifically a polyampholyte, Figure 1B.

**Figure 2:**
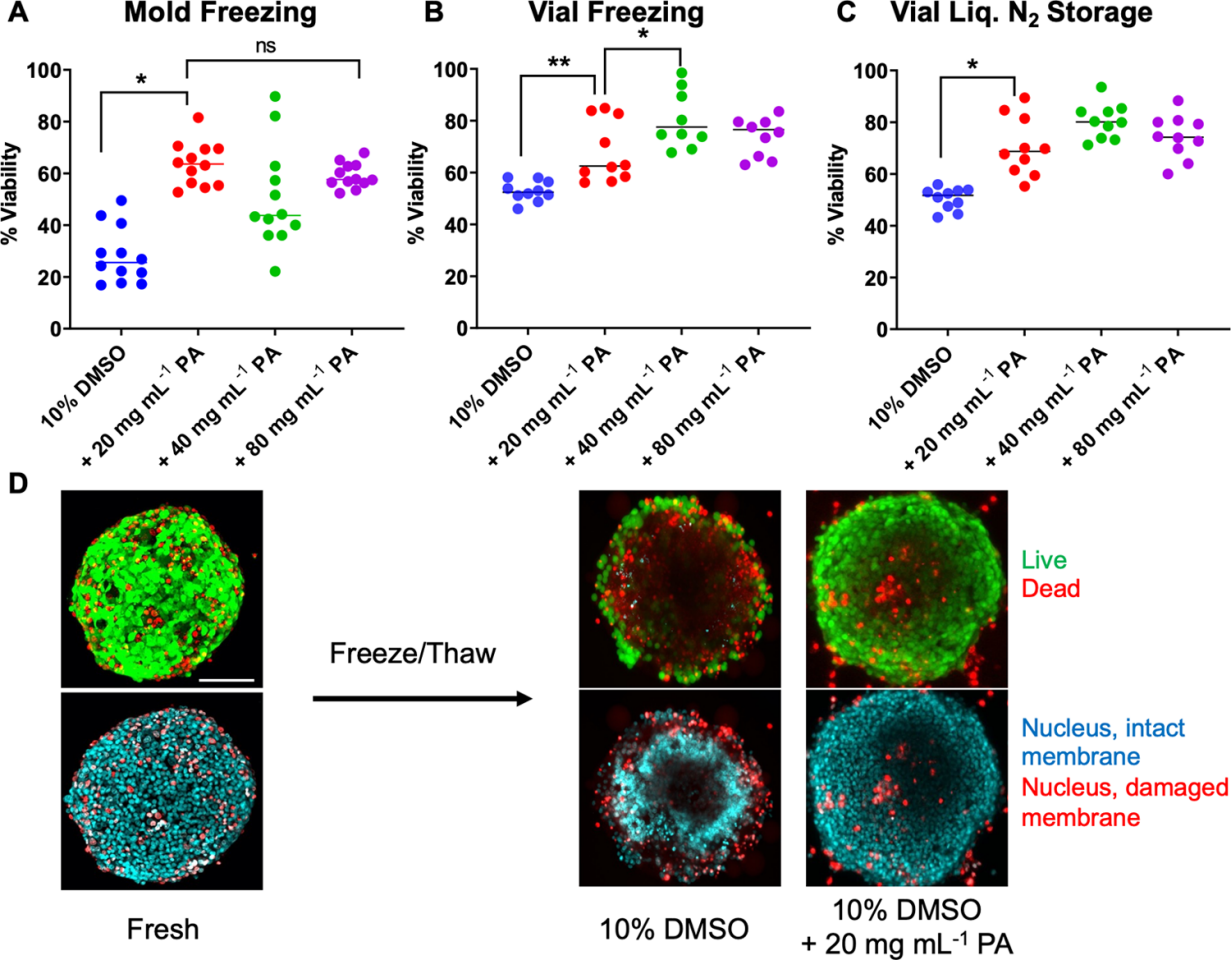
Post-thaw (24 h) recovery of cryopreserved HepG2 spheroids. Percentage viability of spheroids (A) frozen directly in agar molds and stored at −80 °C for 24 h; (B) frozen suspended in cryovials and stored at −80 °C for 24 h; and (C) frozen in cryovials and stored above liquid nitrogen for 3 days. Data are presented as mean % viability relative to pre-frozen spheroids ± SEM from five independent repeats, determined by the WST-1 assay. (D) Confocal microscopy of spheroids before and after thawing in DMSO (10%) with and without supplementation with 20 mg mL^–1^ polyampholyte. Living cells are labeled green (calcein-AM, intact membrane), dead cells are labeled red (EthD-III, damaged membrane), and the nuclei of cells with intact membranes were stained with Hoechst 33342 solution (20 mM). Scale bar = 100 μm.

The as-prepared spheroids were cryopreserved in two formats, either directly within the agar molds or as a suspension in cryovials following their release from the molds at −80 °C. We have previously reported how suspension versus monolayer cell cryopreservation can lead to dramatically different outcomes,^33^ and hence this comparison of freezing formats was important. Figure 2A shows the post-thaw viability of spheroids following cryopreservation with 10% DMSO and a range of concentrations of the polyampholyte (structure shown in Figure 1B). Viability was measured using the WST-1 (metabolic) assay 24 h post-thaw to remove any false positives associated with immediate-post-thaw measurements, which do not account for delayed onset apoptosis.^51^ As expected, due to the confined nature and low total volume of the microwells within the agar molds, the spheroids cryopreserved directly in the molds showed low recovery with DMSO alone (20%), a comparable result to monolayer cryopreservation.^21,45^ The addition of the polyampholyte leads to a significant increase in cell recovery, with 20 mg mL^–1^ being optimal, increasing recovery from ∼30 to ∼60%. Spheroids cryopreserved in vials (suspension) showed higher recovery rates of 50% in DMSO alone, increasing to 75% when the polyampholyte was added. Spheroids were also stored in vials cryopreserved over liquid nitrogen to ensure that they can be banked at temperatures suitable for long-term storage. After 3 days, spheroids were recovered and found to have recovery rates similar to those stored at −80 °C, Figure 2A.

This initial screening demonstrated that the polymers successfully mitigate cryopreservation-induced damage and that they enable the recovery of viable spheroids by simple addition to existing cryopreservation solutions. To determine if cells within spheroids retain membranes intact post-thaw, spheroids were stained with live/dead staining solution before and after freezing and imaged with confocal microscopy, Figure 2B. The live/dead staining confirmed that spheroids cryopreserved with the polyampholyte and DMSO have more intact (green) and fewer damaged (red) cell membranes compared to DMSO alone. Spheroids frozen with DMSO and with or without the polyampholyte were also stained with Hoechst and ethidium iodide (EthD-III) to compare the number of nuclei in membrane damaged (ethidium iodide, red) versus membrane intact (Hoechst, blue) cells, a further comparison of post-thaw membrane integrity and, thus, cellular health. Spheroids cryopreserved with the polymer presented more nuclei stained with Hoechst compared to DMSO alone, where far more EthD-III nuclei were stained, confirming that membrane integrity was rescued. A fundamental biological and biophysical principle of organ regeneration is the tissue fusion. Spheroid fusion can be used to indicate the retention of complex functions not possible with, e.g., monolayer models,^52^ but there is evidence that fatal intracellular ice formation (IIF) can impact the ECM.^53^ Spheroids cryopreserved with/without the polyampholyte were assessed for the spheroid fusion (images in the Supporting Information, Figure S2) showing that the vial-frozen spheroids fused after 7 days, comparable to fresh spheroids, however mold-frozen spheroids, which had lower post-thaw cell viability, fused less. These observations agree with the cell viability measurements that the vial-based freezing is optimal and that the polymer has no negative impact on spheroid function.

**Figure 3:**
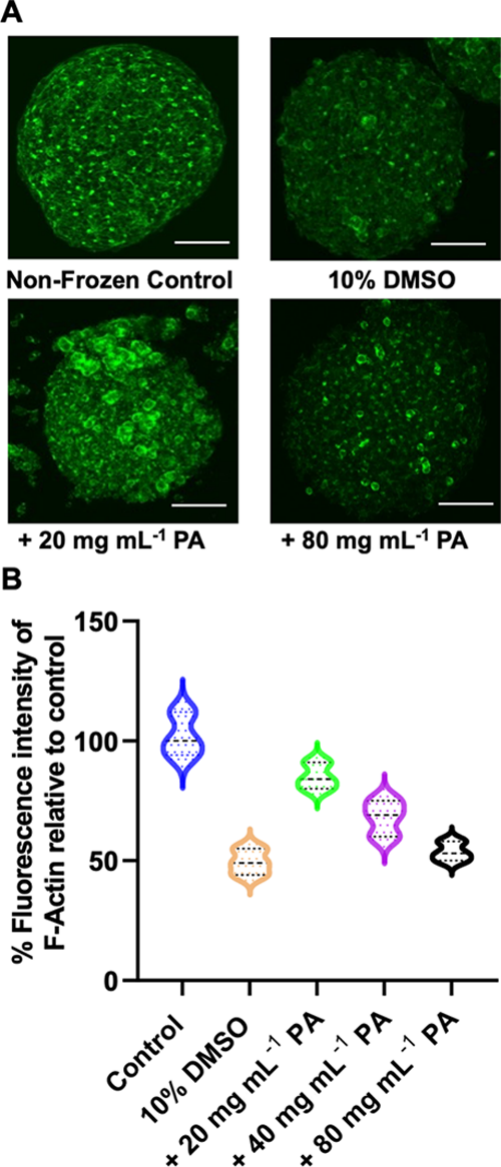
F-actin staining of HepG2 spheroids. (A) Images of HepG2 spheroids pre- and post-thaw (24 h) following cryopreservation using the indicated cryoprotectants; (B) mean fluorescence intensity (MFI) of phalloidin stained F-actin relative to control, non-frozen, spheroids. Data are presented as mean % MFI ± SEM from five independent repeats. Cells were stained for F-actin (phalloidin, green) and nuclei (Hoechst 33342, blue). Scale bars = 100 μm.

F-actin reduction and shortening are associated with cryo-injury, which is problematic due to its critical role in adhesion, migration, proliferation, differentiation,^54^ and maintaining the integrity of cells.^55^ The extent of F-actin depolymerization is dependent on the cooling rate and is important to explore in spheroid cryopreservation. To probe this, spheroids cryopreserved in vials were thawed and stained with phalloidin (with Alexa Fluor 488) to label polymerized F-actin filaments, Figure 3A, and investigate cytoskeletal integrity. Compared to fresh (non-frozen) spheroids, those cryopreserved in 10% DMSO showed significantly lower levels of staining, whereas the addition of 20 mg mL^–1^ of the polyampholyte leads to increased fluorescence and more extensive staining. Polyampholytes have been shown to aid cellular dehydration during freezing (indicated by cell shrinkage),^45^ which in turn modulates the formation of intracellular ice^48,56^ that could impact the extended cytoskeleton network, hence providing a hypothesis for how the polymers rescue actin polymerization post-thaw. The use of 40 mg mL^–1^ polyampholyte (images in the Supporting Information, Figure S7) was also able to preserve cytoskeletal integrity; however, higher concentrations of 80 mg mL^–1^ resulted in diminished actin staining, consistent with the lower recoveries seen in the dose–response data, Figure 2A. Thus, excess polymer fails to further increase post-thaw recovery, as has been previously shown,^42^ and can result in cytoskeletal damage. Cryomicroscopy imaging of HepG2 cell monolayers suggests that polyampholyte reduces intracellular ice formation compared to DMSO alone during the freezing process (Figure S10, the Supporting Information). Intracellular ice growth is observed by the darkening of the cytosol, which was far greater in DMSO alone frozen samples. However, in this experiment, nucleation onset was ∼−20 °C, whereas nucleation of larger volumes, such as those in vials, would occur at much warmer temperatures.^33,34^ Thus, nucleation was mechanically induced at – 8 °C to attempt to mimic the conditions in vial freezing; however, no intracellular ice growth was observed, Figure S11. Hence, further study is required to determine if the polymer modulates intracellular ice formation under the exact spheroid freezing conditions due to this mismatch of nucleation temperatures. Cryomicroscopy was also attempted on intact spheroids during freezing within their molds (which give poor recovery) and those in vials (higher recovery) showing that freezing and IIF occur at higher temperatures (see the Supporting Information, Figures S12 and S13) in vials compared to molds. This correlates with emerging evidence that induced nucleation benefits smaller volumes (i.e., in molds) compared to vials (mL scale).

Flow cytometry was used to determine the population of cells within the spheroid in each cell cycle phase.^45^ As a control, the cell cycle of HepG2 cells in monolayers and spheroids was compared. A large increase in cells within the G0/G1 phase was noted, consistent with an increase in quiescent cells; this is expected as large spheroids (>200 μm in diameter) are formed by concentric arrangements of proliferating cells, intermediate viable cells, quiescent cells, and finally a central necrotic core.^57^ The lower quantity of cells in S and G2-M phases suggested that cells grew and proliferated more slowly in spheroids compared to monolayers.^58^ Following cryopreservation, less cells were observed in the G0/G1 region and, instead, more were found in the S and G2M phases confirming that cells within the spheroids proliferate post-thaw (Table 1).

**Table 1:** Cell Cycle Analysis from Flow Cytometry

	*G*_0_/*G*_1_ (%)	S-phase (%)	G2M (%)
HepG2 monolayer-fresh	65.8	21.5	12.6
HepG2 spheroid-fresh	84.8	3.8	11.0
10% DMSO – post thaw	76.9	5.5	17.6
10% DMSO + 20 mg mL^–1^ PA-post-thaw	80.1	5.7	13.6

**Figure 4:**
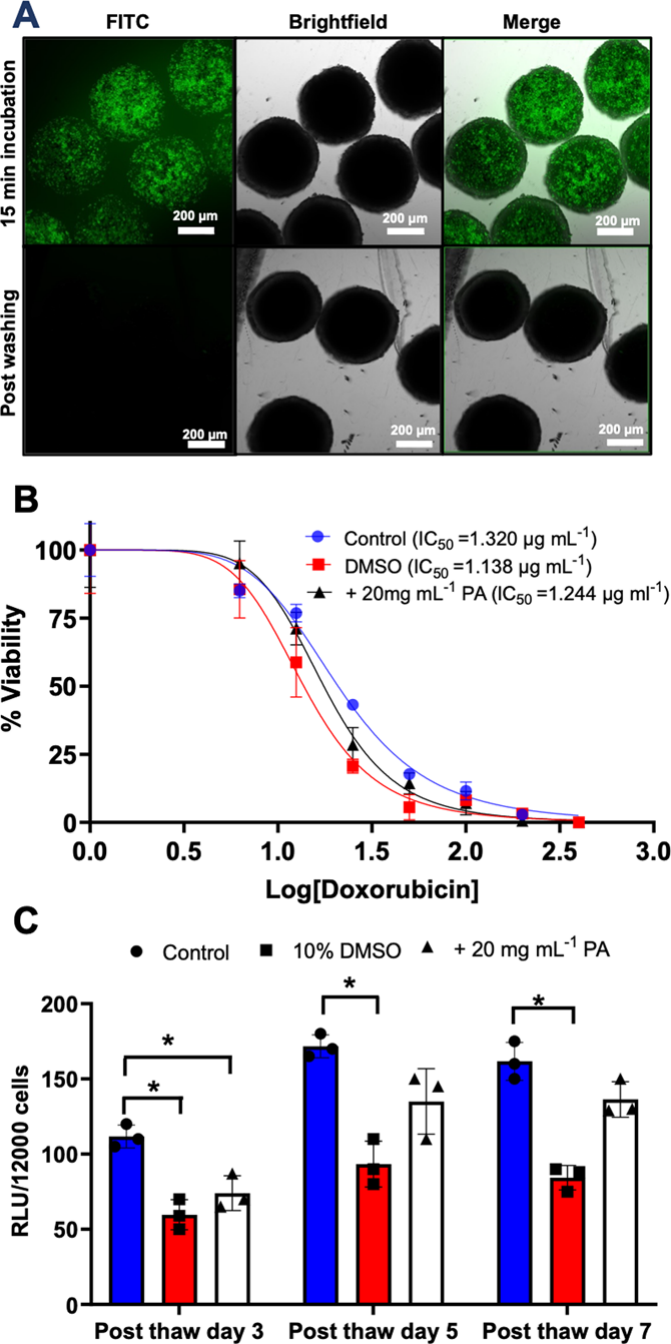
(A) Confocal images of spheroids incubated with the FITC-labeled polyampholyte (15 min) before and after washing. Scale bar = 200 μm. (B) Toxicological challenge of HepG2 spheroids (10 spheroids) against doxorubicin determined by the WST-1 assay. The data are presented as percentage viability relative to doxorubicin untreated spheroids for each freezing condition ± SD for three biological repeats. IC_50_ values are indicated in the legend. (C) HepG2 CYP spheroid Cytochrome P450 3A4 (CYP3A4) activity. CYP3A4 activity was measured before and after freezing at different time intervals post-thaw, having been cryopreserved with 10% DMSO or 10% DMSO combined with 20 mg mL^–1^ of the polyampholyte. The data are represented as mean CYP activity ± SD from three independent repeats. CYP activity is reported in relative light units (RLU) per 12,000 cells (3 spheroids).

For a spheroid to be used in any application, such as toxicity screening, it is desirable to reduce any unwanted interactions during culture by removing the cryoprotectants from cells/spheroids post-thaw. While DMSO must be removed by dilution and washing steps, as it acts intracellularly, the polyampholyte has previously been reported to function extracellularly for cell monolayers, by promoting dehydration^45^ and/or controlling ion flux.^49^ To confirm the extracellular nature of polyampholyte’s mechanism of action, confocal microscopy images of spheroids incubated with the FITC-labeled polymer for 15 min (the same exposure time used during spheroid freezing with the cryoprotectant solution) were taken, Figure 4A. Following washing, negligible cell-associated fluorescence was observed, confirming that the polymer is excluded from the spheroid. The lack of permeation, but protective capability of the polyampholyte, confirms the extracellular mode of action and makes removal of the additives trivial, a key advantage of macromolecular cryoprotectants compared to, e.g., intracellular ice recrystallization inhibitors.^59^

As a functional assay, the dose-dependent toxicological response of cryopreserved spheroids to the chemotherapeutic drug doxorubicin was monitored. Toxicological testing is a primary reason for wanting assay-ready, off-the-shelf, HepG2 spheroids, so it is essential that cryopreserved spheroids can match the function of those prepared fresh. Figure 4B shows the dose–response of fresh, DMSO cryopreserved and DMSO + polymer cryopreserved spheroids treated with doxorubicin. In each case, consistent dose–response curves were seen, with near identical IC_50_ values being obtained. It is important to note, again, that the spheroids cryopreserved with DMSO and the polyampholyte recovered more cells post-thaw and, hence, can be considered a superior cryoprotective solution compared to DMSO alone. The use of cryopreserved spheroids for toxicological assays would be a major benefit in the field of drug discovery, removing the 2 weeks of cell culture preparation normally required to obtain a spheroid, simply by removing it from the freezer.

As a further functional study, the CYP3A4 activity of HepG2 spheroids was investigated post-thaw over 7 days. Cytochromes P450 (CYPs) are a protein superfamily that oxidize steroids, fatty acids, and xenobiotics and are necessary for the clearance of drugs. CYPs are divided into three groups: CYP1, CYP2, and CYP3, with CYP3A4 being the most active and prevalent in human drug metabolism. This isoform may be responsible for more than half of all drug oxidation metabolism reactions mediated by CYP.^60,61^ CYP activity was assessed 3-, 5-, and 7-days post-thaw for spheroids frozen in DMSO and with/without the polyampholyte and compared to fresh spheroids, Figure 4C. In all cases, fresh spheroids presented higher CYP activity compared to the cryopreserved spheroids. However, spheroids cryopreserved with DMSO and polyampholyte presented higher CYP3A4 activity compared to spheroids cryopreserved with DMSO alone 5 days post-thaw, with no statistically significant difference to fresh spheroids (although slightly lower). The CYP3A4 activity of spheroids cryopreserved with DMSO alone remained low and unchanged even after 7 days post-thaw. Thus, the polyampholyte can aid in the recovery of both cell function and viability. While the CYP3A4 activity of polyampholyte cryopreserved spheroids was still slightly lower than those of fresh spheroids, the recovery levels obtained, ease of use of cryopreserved spheroids, and their capacity for use in toxicological challenges all validate the use of macromolecular cryoprotectants to significantly improve spheroid cryopreservation.

## Conclusions

Here, we have demonstrated a viable, straightforward, and potent method to enable the cryopreservation of spheroids, exemplified with HepG2 cells, a widely used cell line in toxicological testing. Banking spheroids is currently challenging, so new cryopreservation methods are required to enable widespread use of spheroids and potentially reduce or complement in vivo toxicological studies. A macromolecular cryoprotectant, based on a polyampholyte, was found to be the key cryopreservation additive required to rescue post-thaw cell viability in spheroids, which 10% DMSO alone could not achieve. Confocal microscopy studies demonstrated that spheroids cryopreserved with the polyampholyte have fewer dead cells, more intact membranes and retained cytoskeletal integrity (F-actin content) compared to DMSO alone. The hypothesis for F-actin rescue is that the polyampholytes can promote cellular dehydration and hence modulate intracellular ice formation. Cryomicroscopy showed evidence of reduced intracellular ice formation in cell monolayers, but the nucleation temperature was not identical to in vial freezing. Hence, the exact role of intracellular ice formation will require further study. Confocal imaging revealed that the fluorescently labeled polyampholyte functions in the extracellular environment, so it is easily removed post-thaw, supporting the use of macromolecular cryoprotectants in generating off-the-shelf spheroids. Polyampholyte cryopreserved spheroids matched the performance of fresh spheroids in a model toxicological challenge and CYP3A4 levels remained high 5 days post-thaw. This work demonstrates that macromolecular cryoprotectants may hold the key to the routine cryopreservation of multicellular spheroids, removing a key bottleneck in the adoption of spheroids in basic and translational research by reducing preparation time from 2 weeks to 72 h and increasing availability.

## Supporting Information Available

Materials and methods including polymer synthesis, spheroid fusion assays, cryomicroscopy methods, and additional fluorescence imaging and cryomicroscopy imaging (PDF)
