# Supplementary material for: Cryopreservation of Liver-Cell Spheroids with Macromolecular Cryoprotectants
Source: ACS Appl Mater Interfaces. 2023 Jan 9;15(2):2630–8. doi: 10.1021/acsami.2c18288 (PMC9869333; doi:10.1021/acsami.2c18288)
Supplement: Supplementary file 1 — am2c18288_si_001.pdf [file am2c18288_si_001.pdf]

Electronic Supporting Information For:

Cryopreservation of Liver-Cell Spheroids with Macromolecular Cryoprotectants

Akalabya Bissoyi,[a] Ruben M. F. Tomás,[a] Yanan Gao [a,c], Qiongyu Guo,[c] and Matthew I. Gibson[a,b*]

[a] *Division of Biomedical Sciences, Warwick Medical School, University of Warwick, Gibbet Hill Road, Coventry, CV4 7AL, UK;*

[b] *Department of Chemistry, University of Warwick, Gibbet Hill Road, Coventry, CV4 7AL, UK;*

[c] *Department of Biomedical Engineering, Southern University of Science and Technology, Shenzhen, Guangdong 518055, China*

*Corresponding Author m.i.gibson@warwick.ac.uk

Experimental Section

Additional Methods

Fusion activity of cryopreserved spheroids

Three freeze/thaw HepG2 spheroids, frozen using both methods, were transferred into Corning™ 96-Well Clear Ultra Low Attachment Microplates and cultured with 100 µL of complete growth medium. Spheroids were incubated at 37 °C and 5% CO_2 and imaged on days 0, 1, 3, 5 and 7 post-thaw using an Olympus CX41 microscope to determine spheroid fusion.

Monolayer Cryomicroscopy Imaging

To further study intracellular ice formation, HepG2 cells were plated on coverslips at a density of 1 x 10^5 cells.[2] The coverslip was positioned on a quartz crucible with a cryoprotectant solution (5 mL) containing polyampholyte (40 $mg.mL^{-1}$) and 10% DMSO, which was placed on a Linkam BCS 196 cryostage. To reach equilibrium, cells were incubated at 20°C for 10 min and subsequently frozen at 1 – 5 $°C.min^{-1}$ until -80 °C either with or without manual nucleation at -8°C using a cooling element. Following freezing, the cells were warmed to RT at $20°C.min^{-1}$. Lynksis 32 software was used to edit and control the cryostage parameters and an Olympus CX41 microscope fitted with a UIS-20x/0.45//0/2FN22 lens and a Canon EOS 500D SLR digital camera was used for video recording. Image processing was completed using ImageJ v1.52 (National Institutes of Health, USA).

Spheroid Cryomicroscopy Imaging

Two studies were carried out with cryomicroscopy imaging to study the effects of intracellular ice formation of cells within spheroids in bulk freezing (i.e. suspension freezing) and surface-based freezing. To the centre of a quartz crucible of 15 mm in diameter (Linkam Scientific Instruments, Salford), 100 µL of cell culture media supplemented with 40 $mg.mL^{-1}$ of polyampholyte and 10% DMSO was added. Spheroids of 3000 cells were placed in the CPA solution and the top of the crucible was covered with a coverslip. The crucible was placed on a Linkam BCS 196 cryostage (Linkam Scientific Instruments, Salford), incubated at 20°C for 10 mins to achieve equilibrium and subsequently frozen at 1 $°C.min^{-1}$ until -40 °C. To thaw the cells, the crucible was warmed to RT for 20 min. For surface-based freezing, spheroids were

positioned on a coverslip and placed on a quartz crucible containing 5 µL of cell culture media supplemented with 40 $mg.mL^{-1}$ of polyampholyte and 10% DMSO. The spheroids were frozen using the Linkam cryostage as described above. Lynksis 32 software was used to edit and control the cryostage parameters and an Olympus CX41 microscope fitted with a UIS-20x/0.45//0/2FN22 lens and a Canon EOS 500D SLR digital camera was used for video recording. Image processing was completed using ImageJ v1.52 (National Institutes of Health, USA).

Effect of freezing on cytoskeleton:

Freeze/thaw spheroids, frozen in cryovials as a suspension with a cryoprotective agent (CPA) consisting of either MEM base media supplemented with 10% (v/v) FBS, 10% (v/v) DMSO and varying concentrations of polyampholyte (0 – 80 $mg.mL^{-1}$), were fixed with 2.5% paraformaldehyde for 30 min and then washed three times with PBS. After fixing, spheroids were permeabilized with 0.2% Triton X-100 in PBS for 10 min at RT and stained with Invitrogen ActinGreen™ 488 ReadyProbe™ reagent (0.145 $mg.mL^{-1}$ in PBS) for 1 h. Spheroids were also stained with a Hoechst 33342 nuclear stain (1 $µg.mL^{-1}$) for 20 min and imaged using an Olympus FV3000 confocal microscope. Hoechst-stained nuclear material was excited using a diode laser with 350 nm and 461 nm emission wavelengths. The Alexa Fluor 488-Phalloidin-labeled actin filaments were excited and optically scanned using an argon-ion laser (excitation and emission wavelengths of 499 and 520 nm, respectively).[3] ImageJ was used to analyze the F-actin mean fluorescent intensity.

Polyampholyte Synthesis

Polyampholyte was synthesised as previously described.[1] Poly(methyl vinyl ether-alt-maleic anhydride) with an average Mn ≈ 80 kDa (10 g) was dissolved in THF (100 mL) heated to 50 °C. Dimethylamino ethanol (~10 g) was added in excess. After 30 min, the waxy solid was dissolved in water (100 mL) and left to stir overnight. The remaining THF was removed under vacuum, and the resulting solution was purified in dialysis tubing (Spectra/Por, 12–14 kDa MWCO) for 72 h with 6 water changes. The polyampholyte was freeze dried, yielding an off-white powder.

Synthesis of Fluorescently Labelled Poly(ampholyte)s.

Fluorescently labelled polyampholyte was synthesised as previously described.[1] The polymer (poly(methyl vinyl ether-alt-maleic anhydride), with an average Mn of 80000 Da (300 mg), was dissolved in THF (50 mL) and heated to 50 °C while stirring. Fluorescein isothiocyanate (FITC) (3 mg) and triethylamine (10 mg) were added after dissolution and left for 20 min. Dimethylamino ethanol (2 g) was added in excess forming a pink waxy solid. Following 30 min, the waxy solid was dissolved in water (50 mL) and left to stir overnight. After adding 50 mL of water, the reaction was stirred overnight before being purified by dialysis. The resulting solution was freeze-dried. The remaining THF was removed under vacuum, and the resulting solution was purified in dialysis tubing (Spectra/Por, 12–14 kDa MWCO) for 72 h with 6 water changes. The polyampholyte was freeze dried.

Intracellular Fluorescently Labelled Poly(ampholyte)s uptake

The intracellular uptake of polyampholytes was examined using fluorescent imaging with fluorescently labelled Polyampholytes dye. Briefly, 10-day-old HepG2 spheroids were incubated with 10% (v/v) FBS, 10% (v/v) DMSO with fluorescently labelled polyampholyte 20 mg.mL^{-1} for three different time periods (15, 30, and 45 min). Spheroids were also imaged after washing with DPBS 3 times. The spheroids before being imaged with an Olympus FV3000 confocal microscope.

Additional Data

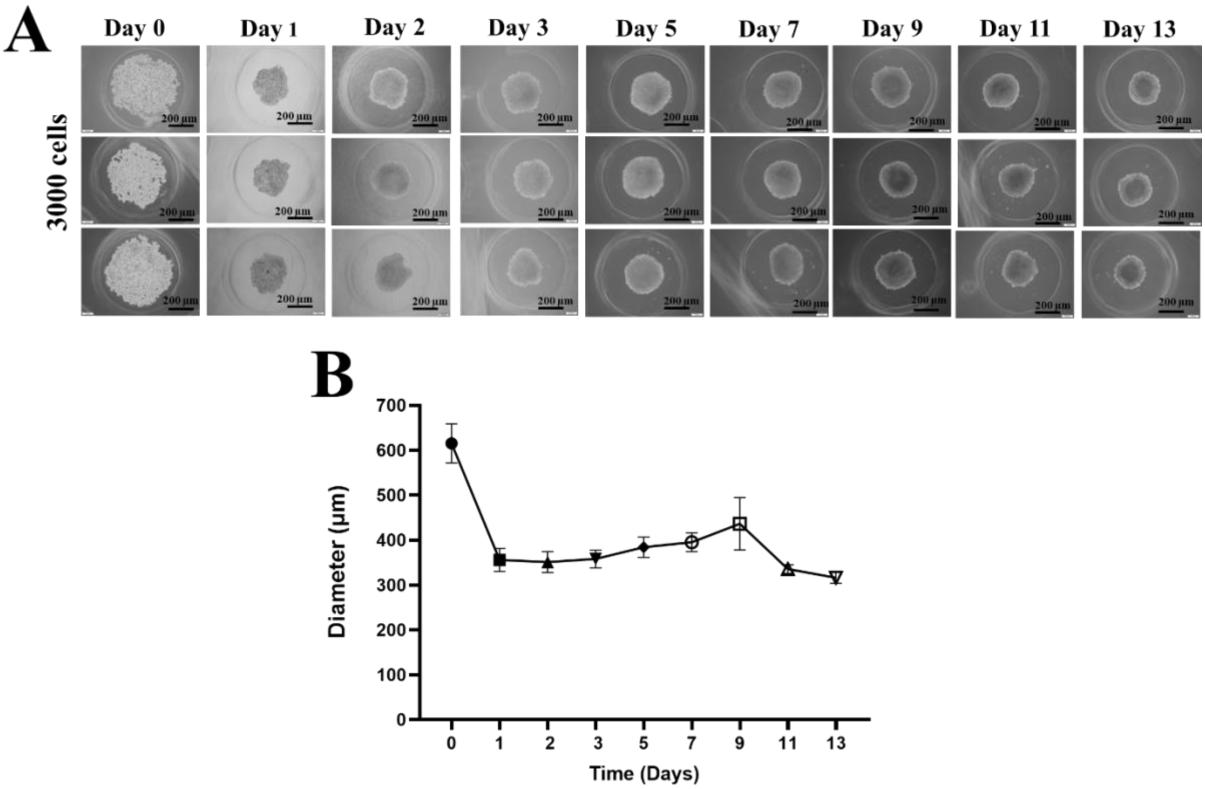

Figure S1. HepG2 spheroid formation and growth using an agar micro mould. (A) Phase-contrast images of HepG2 spheroids (initial seeding density of 3000 cells per spheroid) were recorded on day 0 to day 13 of cultivation. Scale bar = 200 µm. (B) Diameter measurements were obtained from the phase contrast images. Data is represented by mean spheroid diameter ± SD (n = 3).

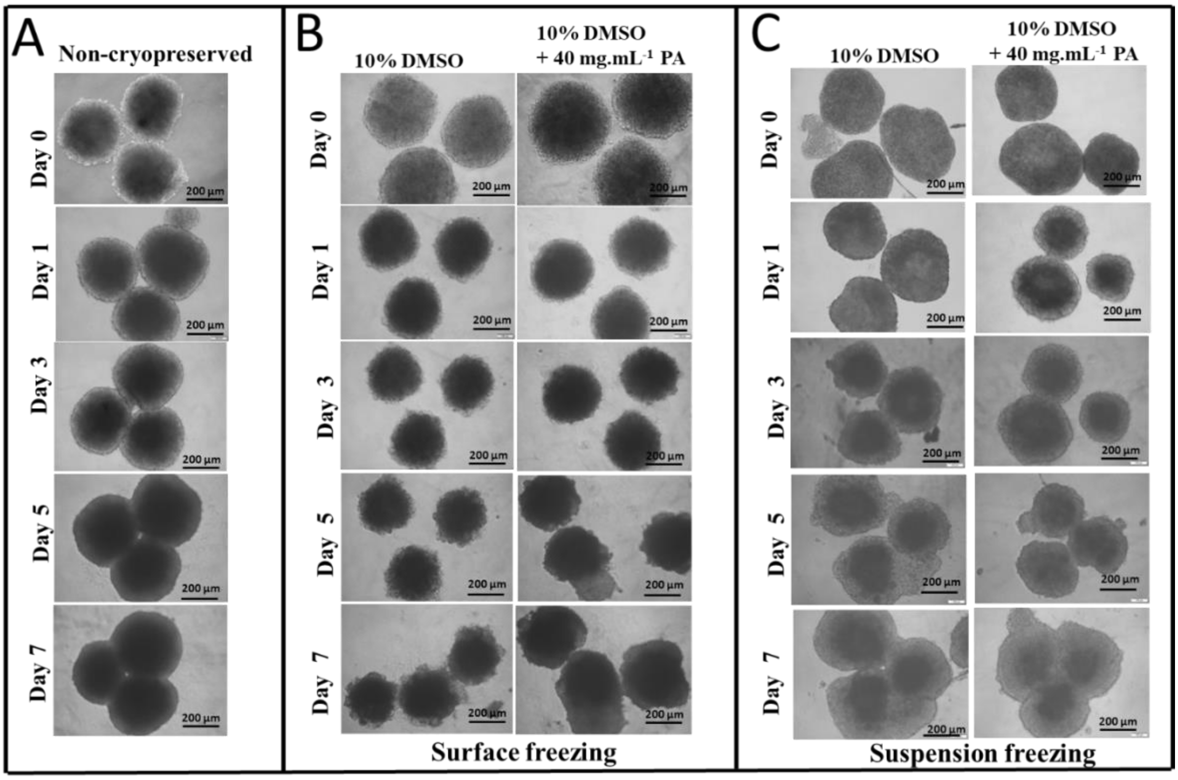

Figure S2. Monitoring the dynamics of spheroid fusion over time in non-frozen and freeze/thawed spheroids. Phase contrast images were recorded of (A) non-cryopreserved HepG2 spheroids, (B) HepG2 spheroids cryopreserved in agar moulds and (C) HepG2 spheroids cryopreserved in cryovials. Cryopreservation was completed using 10% (v/v) DMSO and 40 mg.mL^{-1} of polyampholyte. Scale bar = 200 μm

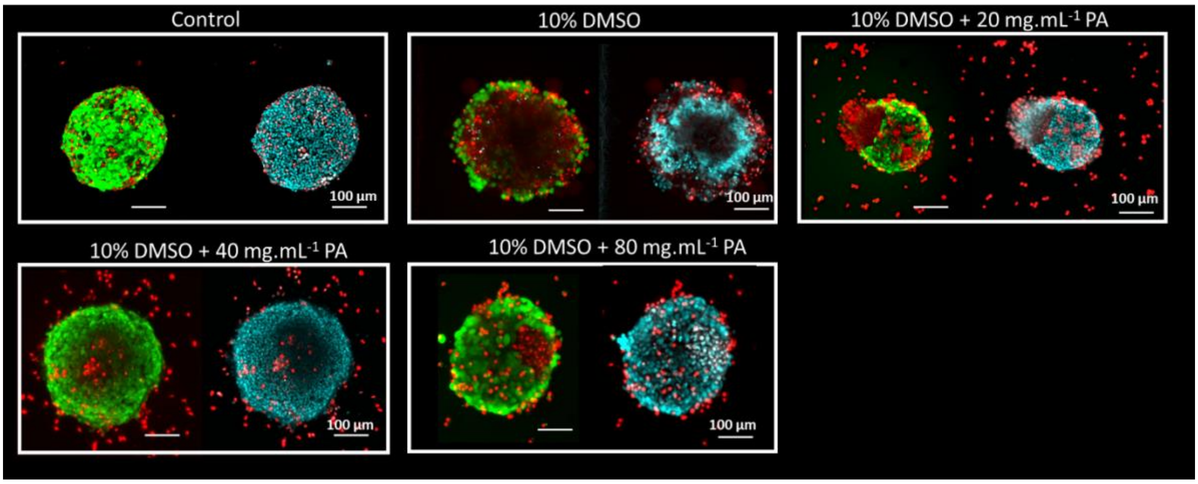

Figure S3. Post-thaw membrane integrity imaging of cryopreserved HepG2 spheroids, 24 h post-thaw. HepG2 spheroids were cryopreserved in 10% DMSO and polyampholyte (PA, 0 – 80 mg.mL^{-1}), thawed and stained with calcein-AM (2 µM, green, membrane intact), ethidium iodide (3 µM, red, membrane damaged) and Hoechst 33342 solution (33 µM, blue, intact nuclei) 24 h post-thaw. Scale bar = 100 µm.

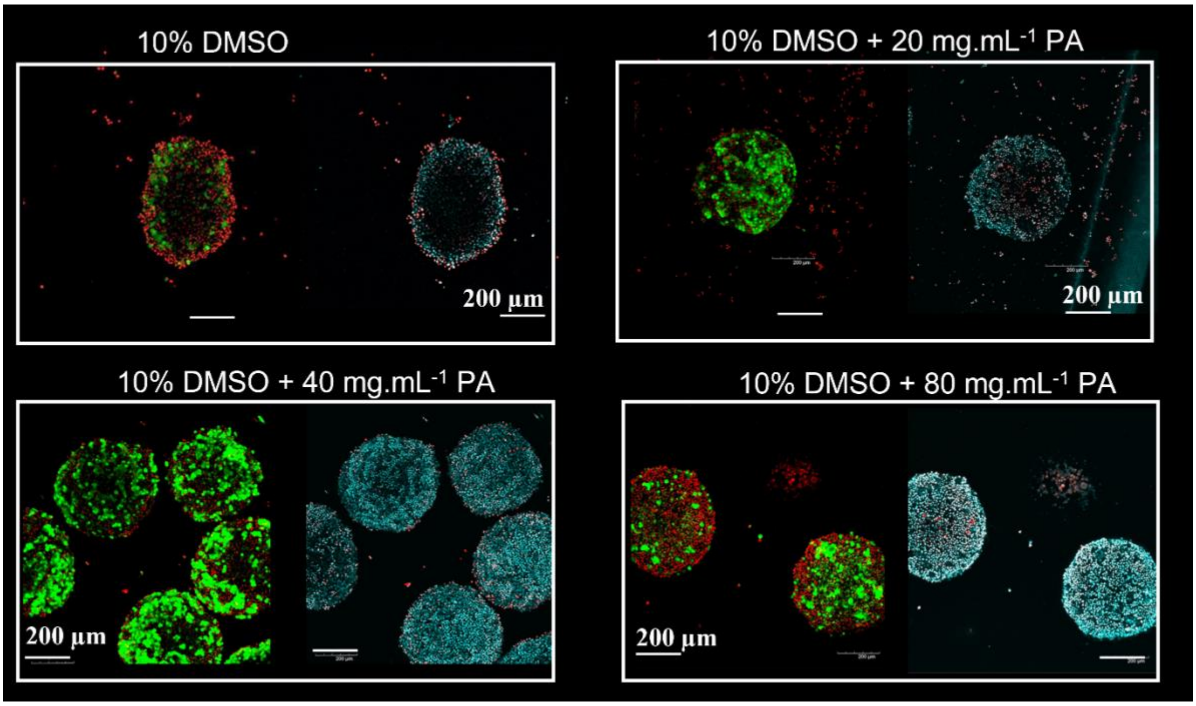

Figure S4. Post-thaw membrane integrity imaging of cryopreserved HepG2 spheroids, 24 h post-thaw after 3 days of liquid nitrogen storage. HepG2 spheroids were cryopreserved in 10% DMSO and polyampholyte (PA, 0 – 80 mg.mL^{-1}), thawed and stained with calcein-AM (2 µM, green, membrane intact), ethidium iodide (3 µM, red, membrane damaged) and Hoechst 33342 solution (33 µM, blue, intact nuclei) 24 h post-thaw. Scale bar = 200 µm.

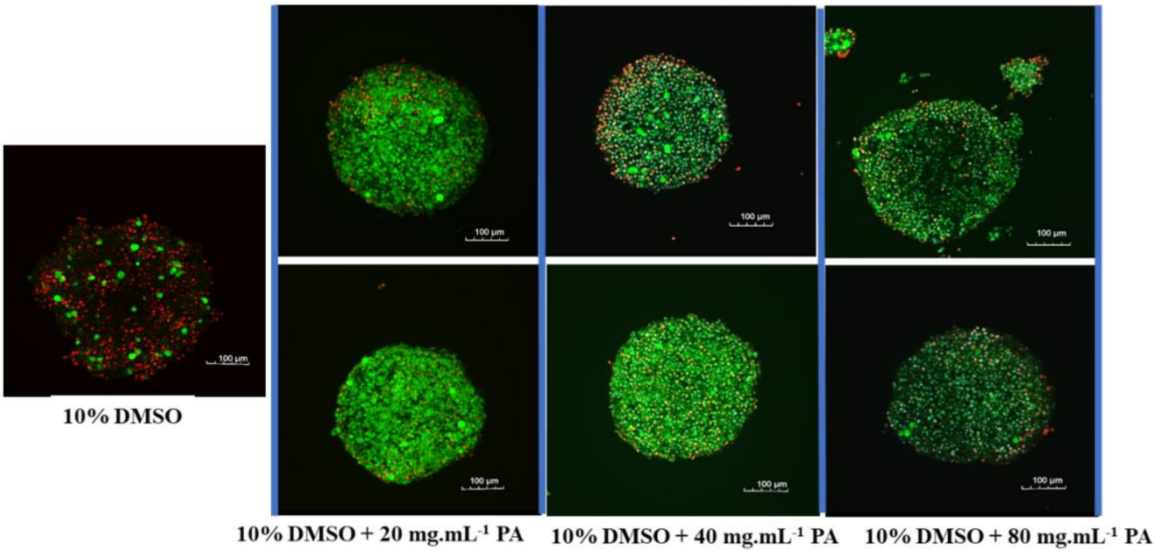

Figure S5. Post-thaw membrane integrity imaging of cryopreserved HepG2 spheroids, 48 h post-thaw. HepG2 spheroids were cryopreserved in 10% DMSO and polyampholyte (PA, 0 – 80 mg.mL^{-1}), thawed and stained with calcein-AM (2 µM, green, membrane intact) and ethidium iodide (3 µM, red, membrane damaged) 48 h post-thaw. Scale bar = 100 µm.

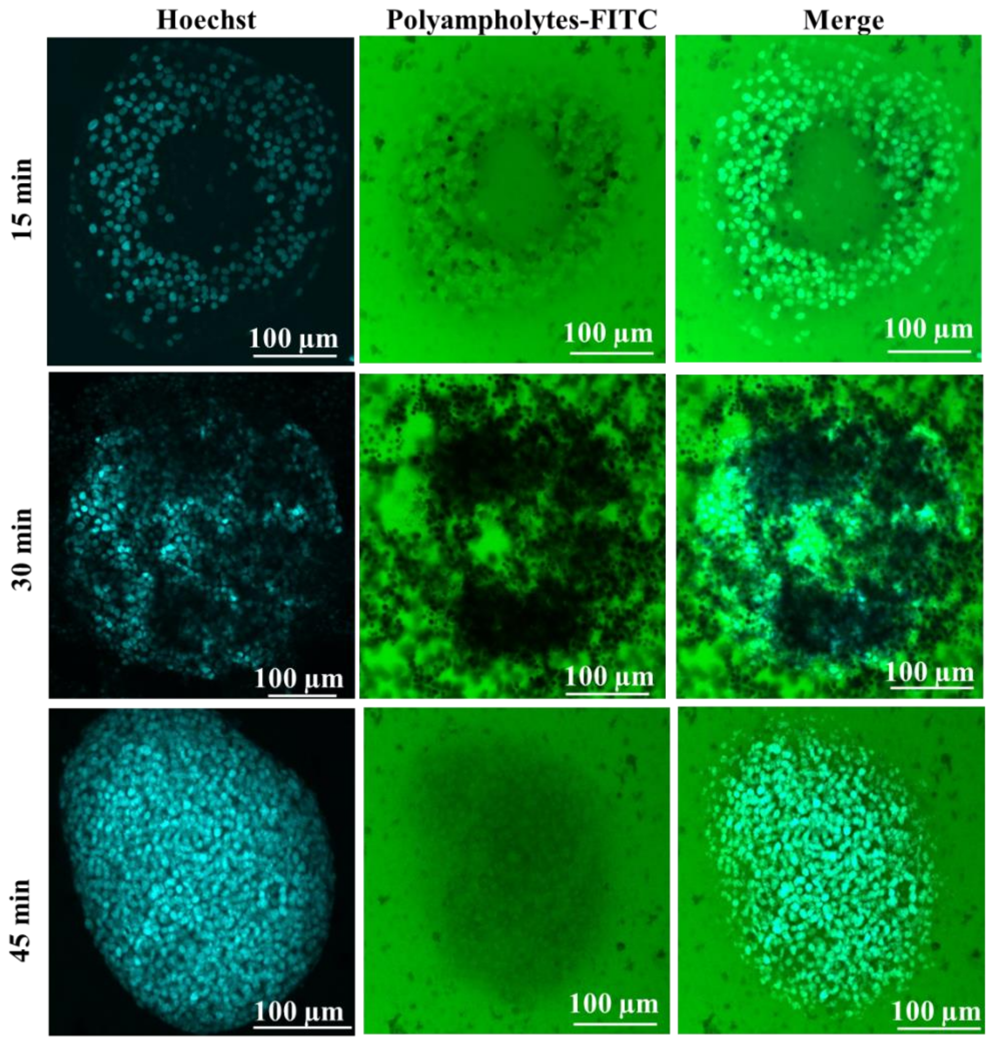

Figure S6. Monitoring the uptake of FITC-labelled polyampholyte in Hepg2 spheroids. HepG2 spheroids were imaged following incubation with FITC-polyampholyte for 15, 30 and 45 min. Scale bar = 100 µm.

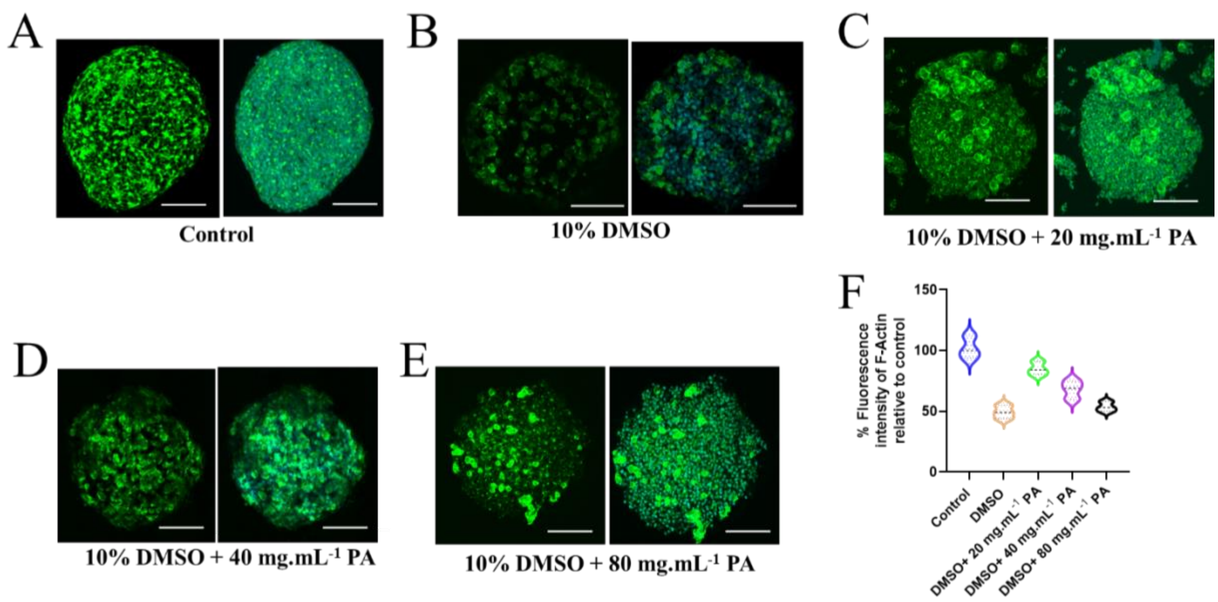

Figure S7. Effect of cryopreservation on cellular cytoskeleton integrity. (A) non-frozen control spheroids and spheroids cryopreserved in (B) 10% DMSO, (C) 10% DMSO and 20 mg.mL^{-1} polyampholyte, (D) 10% DMSO and 40 mg.mL^{-1} polyampholyte and (E) 10% DMSO and 80 mg.mL^{-1} polyampholyte were stained with Invitrogen ActinGreen^{TM} 488 ReadyProbe^{TM} reagent (0.145 mg.mL^{-1}, green, cytoskeleton) and Hoechst 33342 solution (33 µM, blue, intact nuclei) 24 h post-thaw (2 images corresponding to 2 biological repeats). Scale bars 100 µm. (F) The percentage fluorescence of F-actin of the cryopreserved samples was plotted relative to the non-frozen control spheroid samples. Data is presented as %MFI ± SEM from five independent repeats.

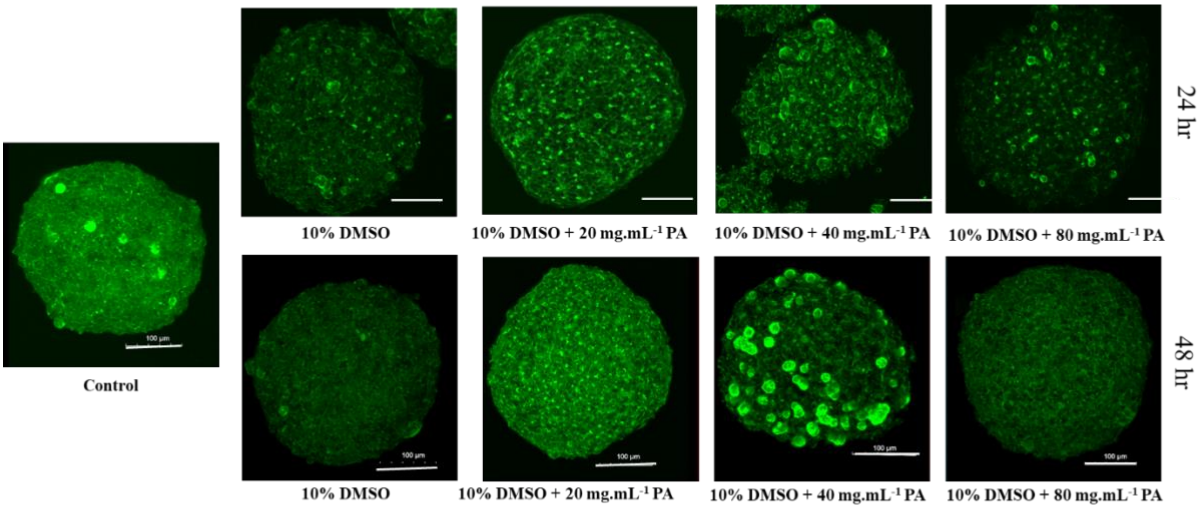

Figure S8. Additional images on the effect of cryopreservation on cellular cytoskeleton integrity. Non-frozen control spheroids and spheroids cryopreserved in 10% DMSO, 10% DMSO and 20 mg.mL^{-1} polyampholyte, 10% DMSO and 40 mg.mL^{-1} polyampholyte and 10% DMSO and 80 mg.mL^{-1} polyampholyte were stained with Invitrogen ActinGreen^{TM} 488 ReadyProbe^{TM} reagent (0.145 mg.mL^{-1}, green, cytoskeleton) 24 h and 48 h post-thaw. Scale bars 100 µm.

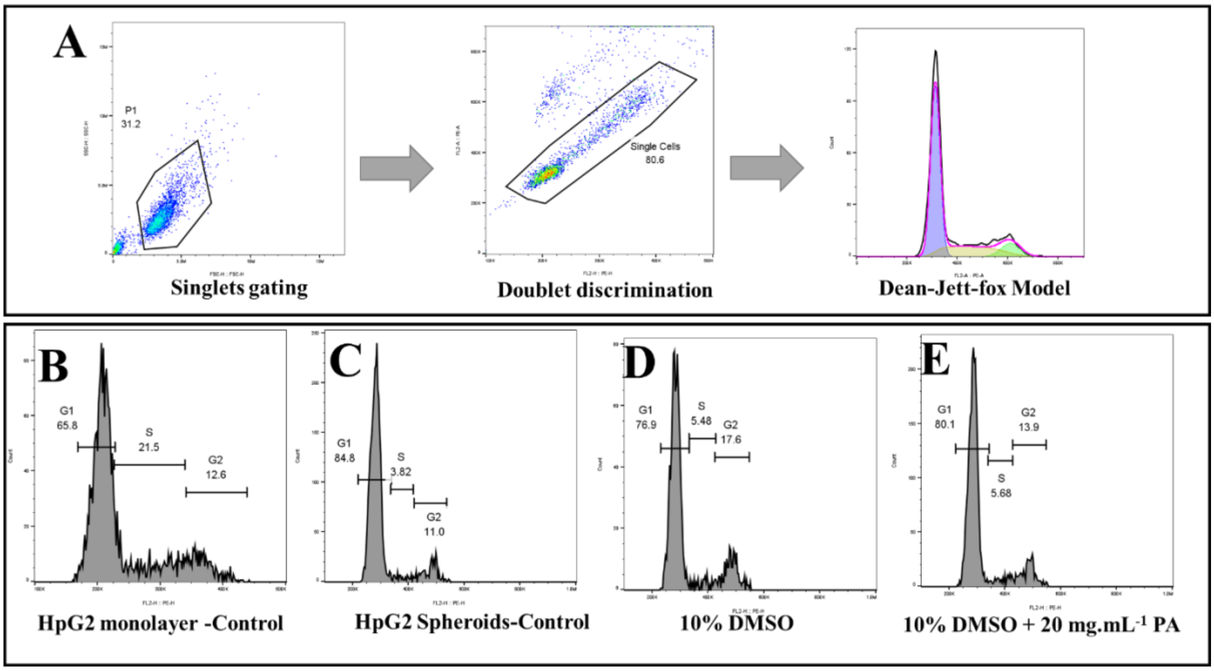

Figure S9. Cell cycle analysis using flow cytometry. (A) General gating workflow of cell cycle analysis. (B) non-frozen HepG2 monolayers, (C) non-frozen HepG2 spheroids and HepG2 spheroids frozen in either (D) 20 mg.mL^{-1} and 10% DMSO or (E) 10% DMSO alone were stained with propidium iodide (20 µg.mL^{-1}) and analysed using flow cytometry.

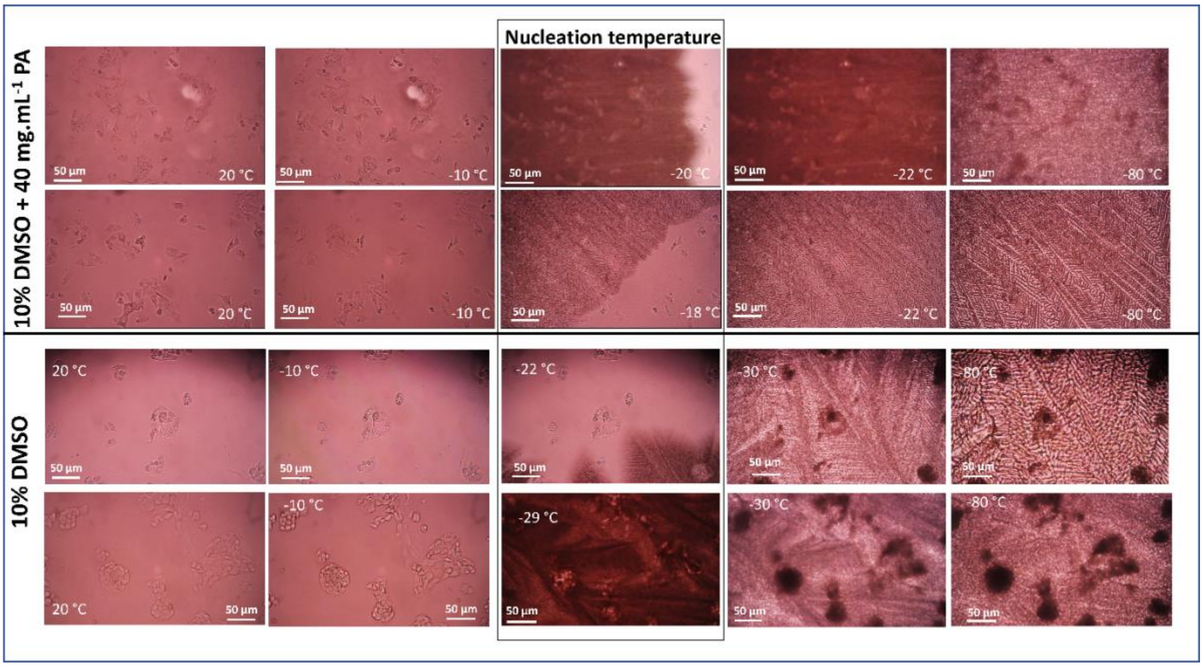

Figure S10. Cryomicroscopy images of HepG2 cells frozen on a cryostage without induced nucleation in the presence of 10 %v/v DMSO and 40 mg. mL^{-1} of polyampholyte to visualise intracellular ice growth. Dark areas highlight regions of ice formation and co-localise with cells. Images were taken at RT (20 °C), -10 °C, -20 °C and -80 °C. Scale bar = 50µm.

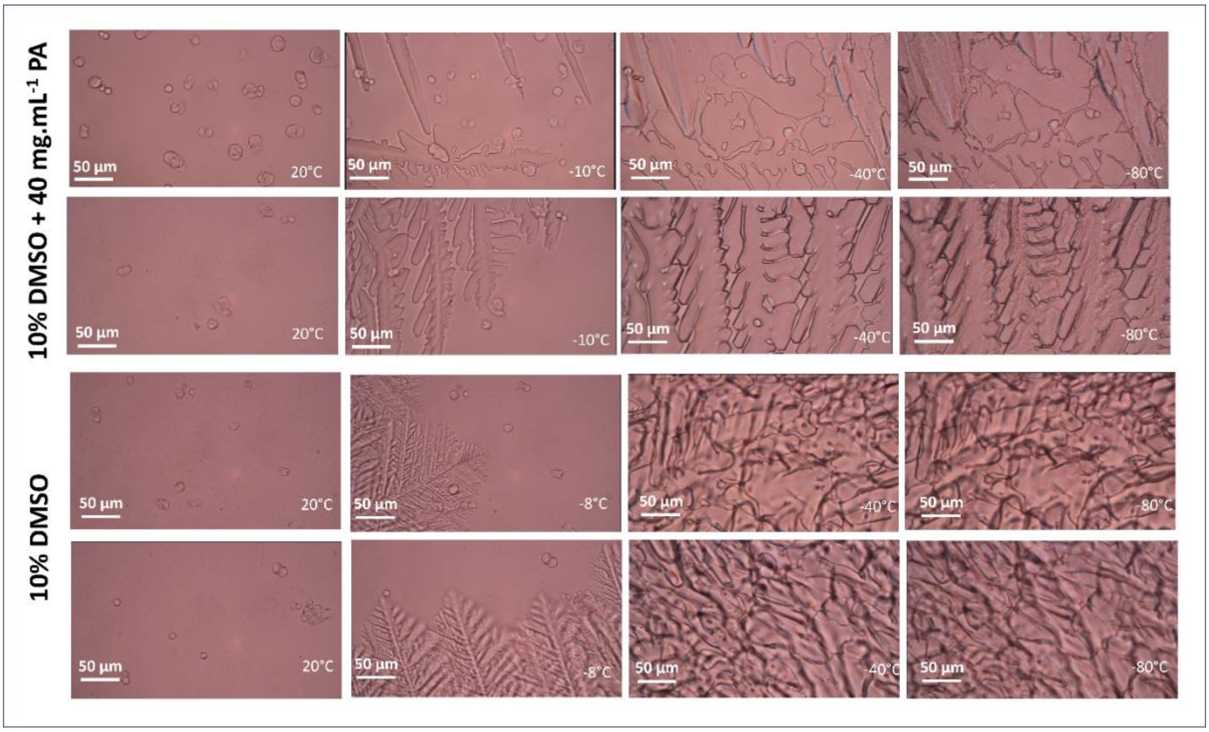

Figure S11. Cryomicroscopy images of HepG2 cells frozen on a cryostage in the presence of 10 %v/v DMSO and 40 mg.mL^{-1} of polyampholyte. Nucleation was mechanically induced at -8 °C to visualise intracellular ice growth. Images were taken at RT (20 °C), -10 °C, -40 °C and -80 °C. Scale bar = 50µm. No ice could be seen in all cases

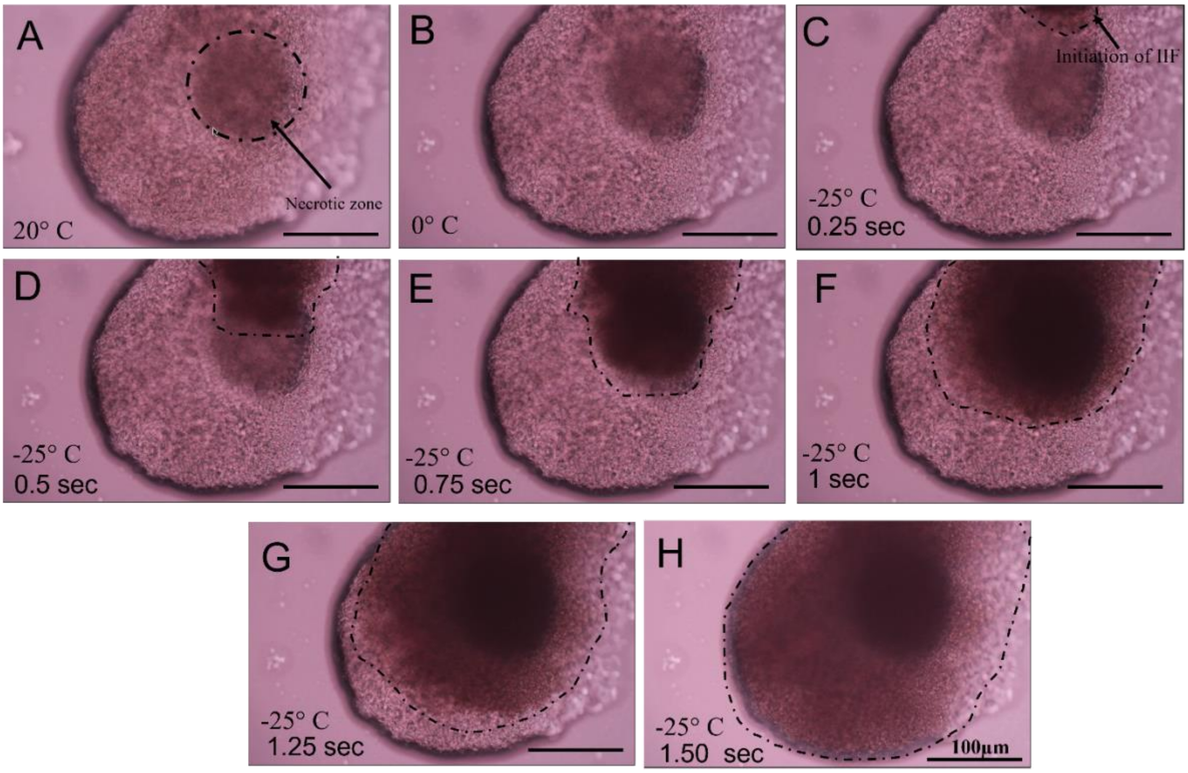

Figure S12. Cryomicroscopy images of spheroids frozen in agar moulds. Spheroids were cryopreserved at a cooling rate of 1°C.min^{-1} and images were captured at (A) 20 °C and (B) 0 °C and (C) 0.25 sec, (D) 0.50 sec (E) 0.75 sec (F) 1.00 sec (G) 1.25 sec (H) 1.50 sec following the formation of intracellular ice formation, visible by cytosolic "darkening", which is initiated at -25 °C. Scale bar = 100 μm.

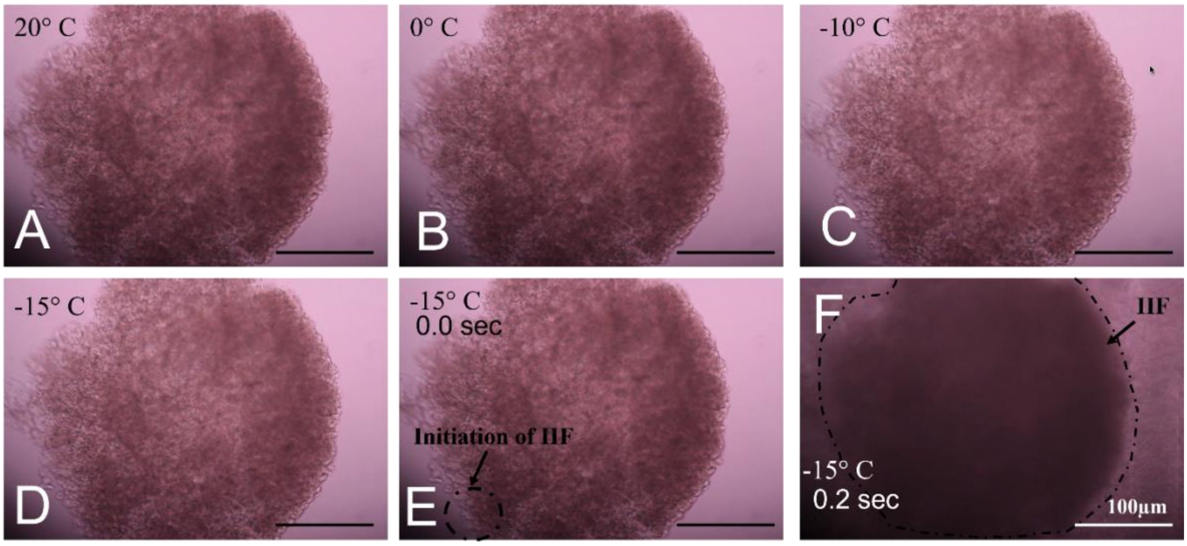

Figure S13. Cryomicroscopy images of spheroids frozen in cryovials. Spheroids were cryopreserved at a cooling rate of 1°C.min^{-1} and images were captured at (A) 20 °C, (B) 0 °C, (C) -10 °C and (D) -15 °C. Images were also captured (E) 0.0 sec and (F) 0.2 sec following the initiation of intracellular ice formation, visible by cytosolic "darkening", at -15 °C. Scale bar = 100 µm.

References

(1) Bailey, T. L.; Stubbs, C.; Murray, K.; Tomas, R. M. F.; Otten, L.; Gibson, M. I. A Synthetically Scalable Poly(Ampholyte) Which Dramatically Enhances Cellular Cryopreservation. *Biomacromolecules* **2019**, *20*, 3104–3114.

(2) Tomás, R. M. F.; Bissoyi, A.; Congdon, T. R.; Gibson, M. I. Assay-Ready Cryopreserved Cell Monolayers Enabled by Macromolecular Cryoprotectants. *Biomacromolecules* **2022**, *23*, 3948–3959.

(3) Chinnadurai, R.; Garcia, M. A.; Sakurai, Y.; Lam, W. A.; Kirk, A. D.; Galipeau, J.; Copland, I. B. Actin Cytoskeletal Disruption Following Cryopreservation Alters the Biodistribution of Human Mesenchymal Stromal Cells in Vivo. *Stem Cell Reports* **2014**, *3* (1), 60–72.
